# Clinical application of single nucleotide polymorphism array in prenatal diagnosis: Experience with 8753 samples

**DOI:** 10.1371/journal.pone.0332424

**Published:** 2025-09-18

**Authors:** Li Wen, Yanzhen Zhang, Wen Zhang, Aifen Mao, Xiangjuan Li

**Affiliations:** Department of Prenatal diagnosis and screening center, Hangzhou Women’s Hospital (Hangzhou Maternity and Child Health Care Hospital), Hangzhou, Zhejiang, China; Universita degli Studi di Roma Tor Vergata, ITALY

## Abstract

**Background:**

Single nucleotide polymorphism (SNP) array has been applied to prenatal diagnosis. This study aimed to assess the clinical significance of SNP array in diagnosing fetal chromosomal anomalies based on our experience.

**Methods:**

A total of 8753 pregnant women with high-risk prenatal indications were enrolled in this retrospective study. Demographic characteristics, chromosomal abnormalities and follow-up results were collected.

**Results:**

Data showed that the overall abnormality detection rate of SNP array was 16.9%, including the detection rate of 7.7% in aneuploidy, the detection rate of 4.2% and 4.4% in pathogenic copy number variations (CNVs) and variants of uncertain significance. The total abnormality detection rate was 15.3%, 22.0% and 38.4% for pregnant women with single risk indication, two kinds of indications and more than two kinds of indications, respectively. The detection rates in the three groups were statistically significant. Additionally, fetus with the positive noninvasive prenatal testing (NIPT) results had the highest abnormality detection rate (38.8%), followed by participants with the abnormal ultrasound findings (13.1%). 98.8% of pregnant women simultaneously chose traditional karyotyping and got their karyotype results. The concordant rate between karyotyping and SNP array was 89.0%, with some structural abnormalities and low-level mosaicism being missed by SNP array, while SNP array additionally found the microduplication/microdeletion.

**Conclusions:**

SNP array would be a valuable technology in prenatal diagnosis, with different abnormality detection rate in pregnant women with various risk indications. SNP array combined with the traditional karyotyping could provide more information for prenatal diagnosis.

## Background

Prenatal diagnosis ranks among the most significant accomplishments in the realm of modern perinatology. The primary indications for invasive prenatal diagnosis consist of high-risk maternal serum screening (MSS), non-invasive prenatal testing (NIPT)-positive results, advanced maternal age (AMA), abnormal ultrasound findings (including structural anomalies and ultrasound soft markers), adverse pregnancy history and chromosomal abnormalities in couples. Since the 1970s, methods of chromosome evaluation that utilize chorionic villi, amniotic fluid and cord blood have been extensively applied and these methods have long been regarded as the gold standard in the diagnosis of fetal abnormalities [1]. Over the past several years, the scope of prenatal diagnosis has broadened. It has evolved from relying solely on karyotyping to incorporating chromosomal microarray analysis (CMA). Standard G-banded karyotyping, the conventional cytogenetic technique used in prenatal diagnosis, can detect chromosomal aneuploidy abnormalities, structural abnormalities and mosaicism. Beyond traditional chromosomal aneuploidy abnormalities, microdeletion, microduplication and copy-number variations (CNVs) that cannot be detected by karyotyping [2], have emerged as a prominent category of birth defects. The advancement of CMA enables us to detect chromosomal aneuploidy and submicroscopic imbalances across the entire genome [3,4]. As one of the CMA types, single nucleotide polymorphism (SNP) array is also capable of identifying not only triploidy but also regions of heterozygosity (ROH) [5]. These arrays make it possible to achieve high-throughput, high resolution and short detection time, thus enhance the clinical utility.

CMA has been proposed as a first-line prenatal diagnostic method in many developed countries [6,7]. It has been reported in several meta-analyses and systematic reviews that submicroscopic copy number changes with clinical significance were detected in 5.2–10% of fetuses simultaneously possessing ultrasound abnormality and a normal karyotype [8–10]. In addition, a multitude of studies have assessed the use of SNP array in prenatal diagnosis [11–13]. However, these studies mainly focused on fetuses with ultrasound structural abnormalities, and pointed out the abnormal SNP array results were most frequently occurred in some specific organ system, such as the genitourinary system, cardiovascular system and central nervous system [14–16]. Little is known regarding the diagnostic performance of SNP array in relation to comprehensive assessment of different prenatal indications. And previous studies were carried out in different laboratories, with various detection platforms being used and inconsistent reporting criteria being adopted. Here, we present our experience regarding the use of SNP array in 8753 pregnant women with different risk factors, and also explore the differences of diagnostic yield between SNP array and the traditional karyotyping. This study could help provide valuable information for clinicians and pregnant women about the application of SNP array technology for prenatal diagnosis in this region.

## Materials and methods

### Study subjects

The present retrospective analysis was carried out among pregnant women who underwent invasive prenatal diagnosis by CMA at the Hangzhou Women’s Hospital (Hangzhou Maternity and Child Health Care Hospital) during the period from January 2015 to December 2023. Qualified clinicians provided all subjects with detailed information regarding the purpose, benefits, and limitations of the prenatal test. Each of the pregnant women provided her signature on the informed consent form and subsequently underwent interventional prenatal diagnostic puncture procedures. Clinical samples including chorionic villi, amniotic fluid and cord blood were obtained by ultrasound-guided abdominal chorionic villus sampling (CVS), amniocentesis and cordocentesis according to the gestational age. The study was approved by the Institutional Ethics Committee of Hangzhou Women’s Hospital (Hangzhou Maternity and Child Health Care Hospital) and implemented in accordance with the Declaration of Helsinki.

### SNP array analysis

The SNP array analysis was performed on the Affymetrix CytoScan platform (Affymetrix, Santa Clara, CA, USA) in strict accordance with the protocol provided by the manufacturer. DNA from villus, umbilical cord, and amniotic fluid was extracted by making use of a genomic DNA extraction kit (TIANamp Micro DNA Kit, China). Then, a quantity of 250 ng genomic DNA was digested, followed by ligation, amplification, purification, fragmentation, labeling and hybridization to the Affymetrix Cytoscan 750K array, which includes 550,000 CNV markers and 200,000 SNP markers. Once the arrays had been washed and scanned, the raw data was analyzed using the Chromosome Analysis Suite (ChAS) software (Affymetrix, Santa Clara, CA, USA) with GRCh37/hg19 assembly. The results were classified according to genetic mode (familial or de novo), CNV length, genes involved, their classification, and literature information. The publicly available databases were utilized as reference resources, including DGV (http://dgv.tcag.ca/dgv/app/home), DECIPHER (https://www.deciphergenomics.org/), OMIM (http://www.omim.org), ClinGen (https://search.clinicalgenome.org/), UCSC (https://genome.ucsc.edu/index.html), Clinvar (http://www.Clinicalgenome.org/data-sharing/Clinvar/) and PubMed (http://www.ncbi.nlm.nih.gov/PubMed). We then used the clinical significance guidelines for CNV developed by the American College of Medical Genetics and Genomics [17,18] to divide our results

into three categories and five levels: (1) pathogenic CNV (pCNV) (including pathogenic and likely pathogenic CNV), (2) variants of uncertain significance (VUS) (3) normal (including benign and likely benign CNV). Mosaicism exceeding 30% and regions of heterozygosity (ROH) with a size larger than 10 Mb were also reported. The median time required for the performance of SNP array analysis was 10 days.

### Statistical analysis

Statistical Product and Service Solutions (SPSS) software version 22 (SPSS Inc., Chicago, USA) was utilized for the statistical analysis. Categorical variables were presented as numbers (percentages) and the chi-square test was applied for comparisons between groups. *P* < 0.05 was considered statistically significant.

## Results

### Basic characteristics

From January 2015 to December 2023, a total of 8753 pregnant women underwent invasive prenatal genetic testing at our prenatal diagnosis center with effective SNP array results. Among them, 95.8% of women chose amniocentesis, while 3.9% chose cordocentesis and 0.3% chose chorionic villus sampling. The ages of the pregnant women ranged from 18 to 49 years, and the gestational ages at invasive testing ranged from 12^+1^ to 36^+4^ weeks. Based on the clinical indications, we divided all samples into the following seven groups: (1) high-risk MSS results (14.3%); (2) NIPT-positive results (13.0%); (3) AMA-only (17.0%); (4) abnormal ultrasound findings (24.7%); (5) other single indications (10.0%), including adverse pregnancy history, medication use or toxic exposure during pregnancy, chromosomal abnormalities in couples, in vitro fertilization, abnormal family history/carriers of genetic diseases; (6) two kinds of indications (20.3%); (7) more than two kinds of indications (0.8%).

### SNP array results

There were a total of 16.9% of samples found to have an abnormal result by SNP array, including 7.7% with chromosomal numerical abnormalities, 8.5% with CNVs and 0.7% with ROH (Table 1). Among the cases with chromosomal numerical abnormalities, 4.6% had common autosomal aneuploidy (CAA), 2.9% had sex chromosome aneuploidy (SCA) and 0.3% had rare autosomal aneuploidy (RAA). Of which, trisomy 21 and XXY were the most common types in CAA and SCA, respectively. Among the cases with CNVs, 4.2% were classified as pathogenic/likely pathogenic (P/LP) (S1 Table) and 4.4% were classified as variants of uncertain significance (VUS) (S2 Table). As shown in Table 1, the abnormal detection rate in the group with ≥ two or two kinds of risk indications was significantly higher than that in the group with single risk indications (P < 0.001). The abnormal detection rate in the group with NIPT-positive results was the highest (38.8%), followed by that in the abnormal ultrasound group (13.1%) and high-risk MSS group (11.0%).

**Table 1:** SNP array results of 8753 pregnant women in different clinical indications.

Clinical indications	Number	Normal	Abnormal						
			Total	CAA	RAA	SCA	CNVs (P/LP)	CNVs (VUS)	ROH
**Single indications**	6906 (78.9%)	5847 (84.7%)	1059 (15.3%)	234 (3.4%)	17 (0.2%)	168 (2.4%)	280 (4.1%)	316 (4.6%)	44 (0.6%)
** **High-risk MSS results	1250 (14.3%)	1112 (89.0%)	138 (11.0%)	32 (2.6%)	1 (0.1%)	9 (0.7%)	31 (2.5%)	54 (4.3%)	11 (0.9%)
** **NIPT-positive results	1138 (13.0%)	697 (61.2%)	441 (38.8%)	135 (11.9%)	12 (1.1%)	115 (10.1%)	114 (10.0%)	57 (5.0%)	8 (0.7%)
** **AMA-only	1484 (17.0%)	1360 (91.6%)	124 (8.4%)	21 (1.4%)	2 (0.1%)	14 (0.9%)	28 (1.9%)	55 (3.7%)	4 (0.3%)
** **Abnormal ultrasound findings	2158 (24.7%)	1876 (86.9%)	282 (13.1%)	45 (2.1%)	2 (0.1%)	27 (1.3%)	83 (3.8%)	110 (5.1%)	15 (0.7%)
** **Others	876 (10.0%)	802 (91.6%)	74 (8.4%)	1 (0.1%)	0 (0%)	3 (0.3%)	24 (2.7%)	40 (4.6%)	6 (0.7%)
**Two kinds of indications**	1774 (20.3%)	1383 (78.0%)	391 (22.0%)	152 (8.6%)	5 (0.3%)	74 (4.2%)	83 (4.7%)	62 (3.5%)	15 (0.8%)
**≥ two kinds of indications**	73 (0.8%)	45 (61.6%)	28 (38.4%)	13 (17.8%)	0 (0%)	8 (11.0%)	3 (4.1%)	4 (5.5%)	0 (0%)
**Total**	8753	7275 (83.1%)	1478 (16.9%)	399 (4.6%)	22 (0.3%)	250 (2.9%)	366 (4.2%)	382 (4.4%)	59 (0.7%)

Abbreviations: MSS, maternal serum screening; NIPT, non-invasive prenatal testing; AMA, advanced maternal age; CAA, common autosomal aneuploidy; RAA, rare autosomal aneuploidy;

SCA, sex chromosome aneuploidy; P, pathogenic; LP, likely pathogenic; VUS, variation of uncertain significance; ROH, regions of heterozygous.

### SNP array on high-risk MSS group

SNP-array analysis was conducted on 1250 pregnant women possessing high-risk MSS results. Among which, 11.0% had abnormal findings, including 3.3% with aneuploidy, 6.8% with CNVs, and 0.9% with ROH (Table 2). These samples were further divided into three subgroups based on their risk types, and the vast majority of which were pregnant women with high risk of T21 syndrome, followed by high risk of T18 syndrome and others indicated abnormal single index of MSS (Table 2). According to the statistical results, we have noticed that there were a total of 18 samples diagnosed with trisomy 21 by SNP array, 17 of which came from the group of high risk of T21 syndrome in the maternal serum screening. Meanwhile, samples diagnosed with trisomy 18 were all from the group of high risk of T18 syndrome.

**Table 2:** SNP array results of 1250 pregnant women with high-risk MSS results.

MSS groups	Number	Total normal	Aneuploidy					CNVs (P/LP)	CNVs (VUS)	ROH
			T21	T18	T13	RAA	SCA			
**High risk of T21**	1162 (93.0%)	1049 (90.3%)	17 (1.5%)	0 (0%)	0 (0%)	1 (0.1%)	9 (0.8%)	27 (2.3%)	49 (4.2%)	10 (0.9%)
**High risk of T18**	76 (6.1%)	52 (68.4%)	0 (0%)	13 (17.1%)	1 (1.3%)	0 (0%)	0 (0%)	4 (5.3%)	5 (6.6%)	1 (1.3%)
^a^ **Others**	12 (1.0%)	11 (90.9%)	1 (8.3%)	0 (0%)	0 (0%)	0 (0%)	0 (0%)	0 (0.0%)	0 (0.0%)	0 (0.0%)
**Total**	1250	1112 (89.0%)	18 (1.4%)	13 (1.0%)	1 (0.1%)	1 (0.1%)	9 (0.7%)	31 (2.5%)	54 (4.3%)	11 (0.9%)

^a^Others indicated abnormal single index of MSS.

### SNP array on NIPT-positive group

A total of 1138 pregnant women at high risk of NIPT were provided with SNP array testing, and 38.8% of them were recognized to present chromosome abnormalities, including 23.0% with aneuploidy, 15.0% with CNVs, and 0.7% with ROH (Table 3). According to the different chromosome locations of NIPT-positive results, we divided the samples into five subgroups. Among the patients in the abnormal chr21 group, 110 had fetuses diagnosed with trisomy 21 and 2 had fetuses diagnosed with CNVs on chr21 (1 CNV (P/LP) and 1 CNV (VUS)). SNP array analysis confirmed 20 cases of trisomy 18 and 9 cases of CNVs on chr18 (6 CNVs (P/LP) and 3 CNVs (VUS)) in the abnormal chr18 group. In the abnormal chr13 group, 5 were verified with trisomy 13 and 5 were verified with CNVs (VUS) on chr13. Meanwhile, of the patients presenting abnormal sex chromosomes results by NIPT, 114 fetuses were diagnosed with SCA, 36 fetuses had CNVs (P/LP), and 3 fetuses had CNVs (VUS) on sex chromosomes. As to the pregnant women with NIPT-positive results on other autosomes, chromosome abnormalities were detected in 24.3% of patients, including 2.2% with aneuploidy, 20.5% with CNVs, and 1.6% with ROH. The overall consistency rate between SNP array results and NIPT-positive indications found on other autosomes was 19.5%.

**Table 3:** SNP array results of 1138 pregnant women with NIPT-positive results.

NIPT-positive groups	Number	Total normal	Aneuploidy	CNVs (P/LP)	CNVs (VUS)	ROH
**Chr21**	159	45 (28.3%)	110 (69.2%)	1 (0.6%)	3 (1.9%)	0 (0%)
**Chr18**	71	41 (57.7%)	20 (28.2%)	6 (8.5%)	4 (5.6%)	0 (0%)
**Chr13**	57	43 (75.4%)	7 (12.3%)	1 (1.8%)	6 (10.5%)	0 (0%)
**Sex chromosomes**	349	188 (53.9%)	114 (32.7%)	40 (11.5%)	7 (2.0%)	0 (0%)
^a^ **Other autosomes**	502	380 (75.7%)	11 (2.2%)	66 (13.1%)	37 (7.4%)	8 (1.6%)
**Total**	1138	697 (61.2%)	262 (23.0%)	114 (10.0%)	57 (5.0%)	8 (0.7%)

^a^Other autosomes indicated autosomes except for chr21, chr18 and chr13.

### SNP array on AMA-only group

There were a total of 1484 samples with clinical indications of AMA only, the average age of the pregnant women in this group was 41.1 years old (35–48 years). Chromosome abnormalities were detected in 8.4% of patients, including 2.5% with aneuploidy, 5.6% with CNVs, and 0.3% with ROH (Table 4). We further divided these samples into three subgroups based on their maternal ages. As shown in Table 4, we found that the total abnormal rate (especially the aneuploidy rate) was gradually increased with advancing age, whereas the pathogenic CNVs rate did not.

**Table 4:** SNP array results of 1484 pregnant women with AMA only.

Age groups	Number	Total normal	Aneuploidy	CNVs (P/LP)	CNVs (VUS)	ROH
**35–39**	898	836 (93.1%)	14 (1.6%)	17 (1.9%)	28 (3.1%)	1 (0.1%)
**40–44**	549	492 (89.6%)	20 (3.6%)	10 (1.8%)	25 (4.6%)	2 (0.4%)
**≥45**	37	30 (81.1%)	3 (8.1%)	1 (2.7%)	2 (5.4%)	1 (2.7%)
**Total**	1484	1360 (91.6%)	37 (2.5%)	28 (1.9%)	55 (3.7%)	4 (0.3%)

### SNP array on abnormal ultrasound group

Fetal anomalies were shown on ultrasound scan in a total of 2158 patients, among which 13.1% were detected with chromosome abnormalities (Table 5), including 3.4% with aneuploidy, 8.9% with CNVs, and 0.7% with ROH. Considering the features of ultrasound, these women were grouped into five subgroups. There were 44.7% of cases having abnormality of a single ultrasonic soft marker, 7.1% of cases having abnormality of multiple ultrasonic soft markers, 39.3% of cases having structural abnormality of a single system, 3.5% of cases having structural abnormality of multiple systems, and 5.4% of cases simultaneously having structural abnormality and ultrasonic soft marker. As shown in Table 5, the group with structural abnormality of multiple systems had the highest rate of chromosomal abnormalities detected by SNP array, while it detected the lowest rate of chromosomal abnormalities in the group with abnormality of a single ultrasonic soft marker.

**Table 5:** SNP array results of 2158 pregnant women with abnormal ultrasound findings.

Ultrasonic anomaly	Number	Total normal	Aneuploidy	CNVs (P/LP)	CNVs (VOUS)	ROH
**Abnormality of a single ultrasonic soft marker**	965 (44.7%)	874 (90.6%)	21 (2.2%)	28 (2.9%)	34 (3.5%)	8 (0.8%)
**Abnormality of multiple ultrasonic soft markers**	153 (7.1%)	132 (86.3%)	5 (3.3%)	9 (5.9%)	**7** (4.6%)	0 (0%)
**Structural abnormality of a single system**	849 (39.3%)	741 (87.3%)	31 (3.7%)	28 (3.3%)	45 (5.3%)	4 (0.5%)
**Structural abnormality of multiple systems**	75 (3.5%)	46 (61.3%)	9 (12.0%)	8 (10.7%)	11 (14.7%)	1 (1.3%)
**Structural anomaly combined** **with soft marker abnormalities**	116 (5.4%)	83 (71.6%)	8 (6.9%)	10 (8.6%)	13 (11.2%)	2 (1.7%)
**Total**	2158	1876 (86.9%)	74 (3.4%)	83 (3.8%)	110 (5.1%)	15 (0.7%)

### SNP array on other single indication group

Out of 876 patients having other single indications, SNP array confirmed 8.4% of cases with chromosome abnormalities, including 0.5% with aneuploidy, 7.3% with CNVs, and 0.7% with ROH (Table 1). Adverse pregnancy history was the main risk indication in this group, accounting for 75.3%, and SNP array verified 3 cases with aneuploidy (1 case of 47,XYY and 2 cases of 47,XXX mosaicism), 17 cases with CNVs (P/LP), 32 cases with CNVs (VUS) and 3 cases with ROH.

### SNP array on multiple indications group

In addition, we also analyzed the SNP array results in pregnant women with two kinds of prenatal diagnostic indications and more than two kinds of indications. The abnormal detection rate of single indication was 15.3%, while that of two kinds of indications and more than two kinds of indications were 22.0% and 38.4%, respectively (Table 1). We found that the total abnormal rate was gradually raised as the number of risk indications which a pregnant woman simultaneously possessed increased. This trend is even more evident in the detection of numerical chromosomal abnormalities.

### Follow-up results

All subjects were followed up either by telephone or during outpatient visits, the records showed that 95.6% of cases were successfully followed up. Among the fetuses with aneuploidies, 568 were stillbirth or terminated by the parents. Additionally, 103 fetuses were permitted to continue their gestation, mainly possessing the karyotype of 47,XXX or 47,XYY. As a result, only 1 case of ventricular septal defect with the karyotype of mosaic 47,XXY had been found up to now. Among the fetuses with CNVs (P/LP/VUS), CMA analysis was performed on 42.8% of parents. Of these, 80.3% of patients had parental inheritance, while the remaining had *de novo* mutations. The rate of pregnancy termination was higher in the CNVs (P/LP) group than in the CNVs (VUS) group (79.8% vs 20.4%). Of the fetuses with CNVs (P/LP), 64 pregnant women continued their pregnancy, producing 61 healthy babies and 3 infants with abnormal manifestations (Table 6). The remaining 10 cases refused to follow-up. Of the fetuses with CNVs (VUS), 284 pregnant women opted to continue their pregnancy, resulting in 274 healthy infants and 10 cases of postnatal abnormalities (Table 6). Only 20 of these cases were lost to follow-up. Furthermore, among the fetuses with ROH, 12 were terminated for various reasons, while 44 cases were live born with 1 having congenital heart disease and hemangioma.

**Table 6:** The abnormal pregnancy outcomes of 13 fetuses with CNVs.

Case	Clinical indications	SNP-array results	Size	Parental testing	Postnatal outcome
**CNVs (P/LP)**					
1	Chromosomal abnormalities in couples	arr[hg19] Yq11.223q11.23(24,820,715−28,464,713)x0	3.6Mb	Paternal inherited	Congenital heart disease
2	Advanced maternal age	arr[hg19] Xp22.31(6,455,151−8,143,319)x0	1.6Mb	Maternal inherited	Ichthyosis
3	NIPT-positive results	arr[hg19] 15q11.2(22,770,421−23,625,785)x1	855Kb	*De novo*	Polydactylism
**CNVs (VUS)**					
1	Adverse pregnancy history	arr[hg19] 4q34.3(178,130,290−179,875,565)x3	1.7Mb	Maternal inherited	Congenital biliary atresia
2	Advanced maternal age	arr[hg19] 8p22(16,339,648−18,158,404)x3	1.8Mb	Maternal inherited	Hypospadias
3	Abnormal ultrasound findings	arr[hg19] 5q32(148,430,463−148,717,687)x1	287Kb	Not available	Abdominal abnormality
4	Abnormal ultrasound findings	arr[hg19] 5q23.1q23.2(120,035,723−121,803,337)x1	1.7Mb	Paternal inherited	Ectopic kidney
5	Abnormal ultrasound findings	arr[hg19] 5p12(44,462,634−45,575,179)x3	1.1Mb	Maternal inherited	Congenital heart disease
6	Abnormal ultrasound findings	arr[hg19] 18q12.1(29,554,989−29,905,356)x1	350Kb	Maternal inherited	Equinovarus
7	Abnormal ultrasound findings	arr[hg19] 7p14.3(31,691,570−31,936,532)×3	245Kb	Not available	Hygroma colli
8	NIPT-positive results	arr[hg19] 15q13.3q14(32,444,261−34,028,683)x1	1.5Mb	Maternal inherited	Congenital heart disease
9	Abnormal ultrasound findings	arr[hg19] 6q26(162,789,315−163,524,994)x1	735Kb	Paternal inherited	Congenital heart disease
10	Abnormal ultrasound findings	arr[hg19] Xq28(151,872,967−153,036,452)x3	1.1Mb	Not available	Esophageal leakage

### Comparison between SNP array and karyotyping

Traditional karyotyping was carried out concurrently on 8682 samples within our cohort, 34 of them failed cell culture, therefore 8648 pregnant women simultaneously possessing SNP array results and karyotype outcomes have been included in this analysis (Table 7). Among cases with numerical chromosomal abnormalities identified by karyotyping, SNP array analysis have the same results in 95.6%. The discrepancy between them were all low-level mosaic aneuploidies, including 2 of trisomy 18, 4 of trisomy 13, 1 of trisomy 2, 1 of trisomy 5, 1 of trisomy 9, 2 of trisomy 11, 1 of trisomy 14, 2 of trisomy 20, 15 of 45,X and 1 of 47 XXX. In normal karyotyping cases, 658 abnormal results were detected by SNP array test, including 19 of mosaic RAA or SCA, 54 of ROH and 585 of microduplication/microdeletion. The prevalence of pathogenic CNVs in normal karyotyping was 2.5%. Among 391 cases with structural chromosomal abnormalities revealed by karyotyping, the consistent rate between SNP array and karyotyping was 33.8%, in which 122 cases with balanced structural rearrangement or marker chromosomes and 126 cases with chromosome polymorphisms were not detected by SNP-array as expected (Table 7).

**Table 7:** The comparison between karyotyping and SNP array results in 8648 samples.

Classification	Detected by karyotyping	Detected by SNP array	Concordant rate (%)
**Normal**	7579	6921^a^	91.3
**Numerical chromosomal abnormalities**	678	648 ^b^	95.6
** Trisomy 21**	305	305	100
** Trisomy 18**	76	74	97.4
** Trisomy 13**	18	14	77.8
** 45, X**	71	56	78.9
** 47, XXX**	51	50	98.0
** 47, XXY**	46	46	100
** 47, XYY**	85	85	100
** 48,XXYY**	1	1	100
** 48 XXX, + 18**	1	1	100
** RAA**	24	16	66.7
**Structural chromosomal abnormalities**	265	132^c^	49.8
**Chromosome polymorphisms**	126	0	0
**Total**	8648	7701	89.0

^a^9 cases of mosaic RAA or SCA, 54 cases of ROH and 585 cases of microduplication/microdeletion;

^b^30 cases of low-level mosaic aneuploidy;

^c^122 cases were normal in SNP array analysis, while SNP-array additionally detected 3 case of ROH and 8 cases of microduplication/microdeletion.

## Discussion

In this study, we carried out an investigation into the clinical significance of SNP array technology in prenatal diagnosis with a cohort of 8753 pregnant women. The overall rate of abnormality detection was 16.9%. Among these cases, 7.7% presented numerical chromosome anomalies, 8.5% showed copy number variations, and 0.7% had loss of heterozygosity. When these data were classified based on diverse risk indications, it was observed that the abnormal detection rate in the group with more than two types of indications was higher than that in the group with two types of indications and the single indication group. Meanwhile, fetus with the positive noninvasive prenatal testing (NIPT) results had the highest abnormality detection rate (38.8%), followed by participants with the abnormal ultrasound findings (13.1%). Furthermore, the SNP array detected a total of 4.2% of clinically significant CNVs, namely those classified as pathogenic (P) or likely pathogenic (LP), which was located within the range of 2.4% to 6.8% in other published studies [19–23].

The detection rate of clinically significant CNVs within the NIPT-positive group reached as high as 10.0%, being the highest. The rapid development of massive parallel sequencing has broadened the detection scope of NIPT to include subchromosomal CNVs. Nevertheless, the efficacy of NIPT in the screening of CNVs is yet to be fully developed and remains limited. In 2022, we have assessed the diagnostic accuracy of NIPT in screening for CNVs, and conducted a systematic review and meta-analysis by combining our study results with those reported in other articles. We found that the positive predictive value (PPV) of NIPT in the detection of CNVs was 8.9% in our cohort of 7637 participants and the pooled PPV was 32.86% [24]. By contrast, the clinical performance of NIPT in the detection of common trisomy and SCA was superior [25]. It is worth noting that we are a tertiary referral prenatal diagnosis center, due to the use of different NIPT platforms, which employ varying sequence read depths and algorithms provided by multiple providers, accurate assessment of positive predictive values was not feasible. Due to the technical limitations of NIPT, the detected material is derived from placental cell-free DNA, leading to false-positive results. Other possible factors include early disappearance or cessation of the development of one of the twins, low cfDNA fraction, maternal chromosomal abnormalities and maternal diseases.

In the group with abnormal ultrasound findings, the CNVs (P/LP) rate was identified as the second highest at 3.8%. During prenatal testing, ultrasound scans were being utilized more and more frequently to identify structural abnormalities in the fetus. The enhanced resolution has made it easier to detect minor abnormalities with greater precision. Our study showed that fetuses with a single system structural abnormality had a 3.3% likelihood of carrying a clinically relevant CNV, the likelihood increased to 10.7% for fetuses with structural abnormalities in multiple systems. This was consistent with a systematic review summarized that a causative submicroscopic CNV was detectable in 3.1–7.9% of fetuses with an ultrasound anomaly isolated to one system and in 9.1% of fetuses with multiple ultrasound anomalies [26]. Additionally, in fetuses with chromosomal abnormalities, ultrasound soft markers were frequently observed [27], though controversial whether to perform invasive prenatal diagnosis based on these non-structural anomalies. In our study, the detection rate of CNVs (P/LP) in fetuses with a single ultrasonic soft marker anomaly was 2.9%, while for fetuses with multiple ultrasonic soft marker anomalies, it was 5.9%. Although statistically insignificant (P = 0.056), this increase implied that SNP array should be conducted for pregnant women when ultrasound reveals two or more abnormal soft markers.

Our data further showed that pregnancies with high-risk MSS results had the rate of 2.5% in detecting the clinically significant CNVs. This is in accordance with the finding (2.5%) of a prior study conducted by Xiang et al. [20], and a little higher than the result (1.6%) of a previous NICHD multicenter study [28]. Despite the fact that CNVs (P/LP) detection rate of this group was lower compared to the NIPT-positive group, we cannot directly abandon the MSS test, mainly because it is a more cost-effective option for prenatal screening in our country, and it also identifies high risk of neural tube defects in fetuses.

With the promulgation of the three child policy in our country, the proportion of pregnant women with advanced maternal age has correspondingly increased. In our study, the AMA-only group accounted for 17.0% of the total samples, the detection rates of aneuploidy and CNVs (P/LP) in this group were 2.5% and 1.9%, respectively. Our CNVs (P/LP) detection rate is similar to the 2.35% reported by Li et al. [29] and 1.6% reported by Xiang et al [20]. Subgroup analysis illustrated that there was a notable increase in the rate of numerical chromosome anomalies with increasing age, while the rate of CNVs (P/LP) did not exhibit such a trend. This is in line with the results of earlier research efforts [30,31].

Studies showed that microarray analysis was equivalent to standard karyotype analysis for the prenatal diagnosis of common aneuploidies, while it could provide additional clinically relevant information [28]. In our study, we identified clinically significant CNVs in 4.2% of the cases with the most common effects were 1q21.1deletion syndrome, Williams-Beuren syndrome, 15q11.2 deletion syndrome, Prader-Willi syndrome, 16p11.2 deletion syndrome, 16p13.11 duplication syndrome, hereditary neuropathy with liability to pressure palsies, Holoprosencephaly 4, DiGeorge/Velocardiofacial syndrome, 22q11.2 duplication syndrome, X-linked Ichthyosis (S1 Table). Many of these copy-number variants are typically smaller duplications and deletions than those identified with the use of chromosome banding. If we use the resolution of 5Mb for karyotype analysis, we speculated that SNP array can additionally identify 2.5% of pregnancies with significant microdeletions or microduplications who underwent invasive prenatal diagnosis in our study. These data indicate a benefit to chromosomal microarray analysis as a standard part of prenatal testing.

With the use of SNP array technology, CNVs (VUS) have been detected at the same time (S2 Table). In this study, the detection rate of CNVs (VUS) in the total samples was 4.4%, which is consistent with others describing cases with uncertain significant CNVs account for less than 5% of all cases [32]. Another study from 5026 pregnancies also pointed out 4.6% of cases were detected with variants of uncertain clinical significance [33]. However, the occurrence of VUS during prenatal diagnosis may present challenges for genetic counseling, place pressure on pregnant women and their families, and lead to excessive termination of pregnancy. VUS cases are the most likely to have favorable pregnancy outcomes, methods for the accurate evaluation of these cases needs to be further determined by clinicians. Consequently, cases showing VUS abnormalities necessitate additional follow-up for experience accumulation and better consultation. This study has significant implications for the development of future genetic counseling guidelines.

SNP array is a powerful tool in genetic analysis, especially when it comes to detecting ROH [34]. According to the local expert guidelines, ROH involving chr6, chr7, chr11, chr14, chr15, chr20 with 5 Mb (at the end of the chromosome) or 10 Mb (not at the end of the chromosome) on one of these chromosomes and other chromosomes were reported [35]. As chromosomes 6, 7, 11, 14, 15 and 20 are known to be associated with parental-specific expression genes, further testing is necessary to clarify the diagnosis and distinguish between ROH and UPD. In clinical practice, child-parent trio analysis with the same platform is often required [36]. For other chromosomes, the detection of large-segment ROH suggests a risk of recessive genetic diseases, and further sequencing analysis may be considered to screen for homozygous mutations based on prenatal clinical indications. In this study, we identified 59 cases with ROH, of which 44 cases have been further confirmed to be normal and opted to continue their pregnancies. However, 12 cases selected induced labour, including a case of ROH on the whole chromosome 14, the karyotype of this fetus was 45,X,inv(Y)(p11q11),der(14;14)(q10;q10). Another case with the presence of fragmented ROH in the long arm of chromosome 16 showed intrauterine growth restriction in late pregnancy. There were also 5 cases with ultrasound structural abnormalities at the same time. Two fetuses were found to have multiple large regions of ROH due to consanguineous marriage. And other couples abandoned further diagnosis test and terminated their pregnancies due to personal reasons. Given the intricate pathogenic mechanisms of ROH, it is essential to perform a thorough prognosis evaluation integrating ultrasound results, further genetic test in recessive genes, parents verification, and so on.

The advancement of array-based molecular techniques has enhanced the detection of submicroscopic CNVs, that are not typically observable in karyotyping. Researches have shown the additional value that chromosomal microarray brings to the analysis of affected children [37], stillborn pregnancy [38] and prenatal diagnosis [28]. In the current study, SNP array analysis uncovered 658 (658/7579, 8.7%) additional abnormal results compared to karyotyping, including 19 cases of mosaic RAA or SCA, 54 cases of ROH and 585 cases of microduplication/microdeletion. All the numerical chromosomal abnormalities detected in the karyotyping analysis were in agreement with the SNP array results, barring 30 (30/678, 4.4%) cases of low-level mosaicism. Meanwhile, part of structural chromosomal abnormalities (including balanced chromosomal translocations and inversions) remained undetected by SNP array because of the absence of any change in the quantity of genetic materials. Based on the specific advantages of these two technologies, we proposed that karyotyping and SNP array analysis be applied in combination during prenatal diagnosis.

The phenotype resulting from a susceptibility CNV is unpredictable due to incomplete penetrance and variable expressivity [39,40]. When encountered in the prenatal setting, this increased range of phenotypic features can make genetic counseling challenging, many of these CNVs do not always result in severe impairments. In our study, the most common recurrent microdeletion/duplication syndromes were 15q11.2 deletion syndrome, 16p11.2 duplication syndrome, 16p13.11 deletion syndrome, 16p13.11 duplication syndrome, 17p12 duplication syndrome. We observed that many of these fetuses did not exhibit abnormal ultrasound findings and were therefore retained. Moreover, the proportion of normal fetuses after birth is greater in recurrent microdeletion/duplication syndromes with penetrance of less than 10%. For example, approximately 90% of fetuses with 15q11.2 deletion syndrome and 16p13.11 duplication syndrome have a normal outcome. There is currently controversy regarding whether to report this type of pathogenic CNV, posing significant challenges for genetic counseling.

## Conclusions

In conclusion, our study with a sample size of 8753 showed that the detection rate of chromosome abnormalities by SNP array was 16.9%. The detection rates of total anomalies and clinically significant CNVs were different among pregnancies with diverse prenatal indications. We suggested the combined use of karyotyping and SNP array analysis in prenatal diagnosis for more integral information. When CNVs (VUS) or ROH have been detected by SNP array, genetic counselling about how to interpret the results is critical.

## Supporting information

S1 Table Pathologic, Likely pathologic copy number variations found in fetuses by SNP array. (XLSX)

S2 Table Uncertain significance copy number variations found in fetuses by SNP array. (XLSX)
